# Repriming DNA synthesis: an intrinsic restart pathway that maintains efficient genome replication

**DOI:** 10.1093/nar/gkab176

**Published:** 2021-03-21

**Authors:** Lewis J Bainbridge, Rebecca Teague, Aidan J Doherty

**Affiliations:** Genome Damage and Stability Centre, School of Life Sciences, University of Sussex, Brighton, BN1 9RQ, UK; Genome Damage and Stability Centre, School of Life Sciences, University of Sussex, Brighton, BN1 9RQ, UK; Genome Damage and Stability Centre, School of Life Sciences, University of Sussex, Brighton, BN1 9RQ, UK

## Abstract

To bypass a diverse range of fork stalling impediments encountered during genome replication, cells possess a variety of DNA damage tolerance (DDT) mechanisms including translesion synthesis, template switching, and fork reversal. These pathways function to bypass obstacles and allow efficient DNA synthesis to be maintained. In addition, lagging strand obstacles can also be circumvented by downstream priming during Okazaki fragment generation, leaving gaps to be filled post-replication. Whether repriming occurs on the leading strand has been intensely debated over the past half-century. Early studies indicated that both DNA strands were synthesised discontinuously. Although later studies suggested that leading strand synthesis was continuous, leading to the preferred semi-discontinuous replication model. However, more recently it has been established that replicative primases can perform leading strand repriming in prokaryotes. An analogous fork restart mechanism has also been identified in most eukaryotes, which possess a specialist primase called PrimPol that conducts repriming downstream of stalling lesions and structures. PrimPol also plays a more general role in maintaining efficient fork progression. Here, we review and discuss the historical evidence and recent discoveries that substantiate repriming as an intrinsic replication restart pathway for maintaining efficient genome duplication across all domains of life.

## INTRODUCTION: THE EUKARYOTIC DNA REPLICATION MACHINERY

During the synthesis phase (S phase) of the cell cycle, genome replication is performed by the replisome. This multi-protein complex consists of the major replicative enzymes required to accurately duplicate DNA. Replisome proteins include the DNA polymerases α, δ and ϵ, the Cdc45–MCM–GINS (CMG) DNA helicase complex, as well as additional proteins such as AND-1 (yeast Ctf4), Timeless (Tof1), Claspin (Mrc1), Tipin (Csm3), Topoisomerase I, Mcm10, Replication Protein A (RPA) and FACT (1). Replisome assembly begins in G1 phase with the binding of the minichromosome maintenance (MCM) complex to defined loci known as origins of replication (2). Loading of the MCMs to origins is dependent on prior binding of the Origin Recognition Complex (ORC), comprised of ORC1–6, and the proteins Cdc6 and Cdt1 (3). The MCM replicative helicase is loaded as an inactive, double hexamer structure (4), and is activated when DNA replication begins at the start of S phase (reviewed in (5)). The activation process remodels the MCM complex into two active CMG complexes, one for each direction of synthesis. Encircling each leading DNA strand, the active complex moves away from the centre of the origin and allows for the assembly of the remaining replisome components on the resulting single-stranded DNA (ssDNA) (6).

While the bulk of synthesis is completed by the major replicative polymerases (Pol δ and Pol ϵ), these enzymes lack the ability to initiate DNA synthesis *de novo*. Therefore, a short ribonucleotide primer is required, from which 3′ extension can be continued by the replicative polymerases (7). In the conventional model, the initiating primers on both the leading and lagging strand are generated by the Pol α-primase complex. This primase synthesises a short RNA primer *de novo*, from which Pol α can extend using dNTPs to create an RNA-DNA primer. This is then further extended by a primary replicative polymerase with proofreading capacity, to ensure high fidelity synthesis. Polymerase usage throughout replication is well-coordinated, with the majority of leading strand synthesis undertaken by Pol ϵ, while Pol δ copies the lagging strand (8). However, this may not always be the case, as Pol δ can conduct synthesis on both strands in yeast, both during bulk replication and following replication restart (9,10). All polymerases exclusively synthesise DNA in a 5′ to 3′ direction. For this reason, the lagging strand is synthesised in short, discontinuous fragments, as the DNA is unwound to allow coupled unidirectional replication to occur (11,12). The Pri1/Pri2 (PriS/L) primase complex frequently synthesises ribonucleotide primers on the lagging strand template, from which Pol α and Pol δ can extend (13). The generally accepted model for leading strand synthesis involves continuous synthesis by Pol ϵ from the Pri1/Pri2 generated primer at the origin until termination (14). Pol ϵ is more processive than Pol δ, in keeping with its role of replicating the majority of the leading strand (15,16). Termination of DNA replication occurs either when converging replication forks meet or when the end of the chromosome is reached (17). The replication machinery is then unloaded by the ATPase p97 (cdc48 in yeast), to prevent re-replication of DNA (18). Unlike replication initiation, which is well studied in eukaryotes, replication termination has received significantly less attention. For this reason, the current understanding of replication termination is somewhat incomplete.

### Replication stress: derailing the DNA replication machinery

During genome duplication, the replication fork encounters a myriad of conditions and obstacles that can affect the progression of DNA polymerases, resulting in replication stress. Pol δ and Pol ϵ operate with high fidelity to accurately copy DNA and stall at atypical bases or DNA structures, due to an inability to bypass distorted templates (19). Causes of polymerase stalling include unrepaired DNA lesions generated by both endogenous and exogenous sources (20), DNA secondary structures such as G4 quadruplexes (21) or R loops (22), proteins tightly bound to DNA (23), repetitive sequences, including common fragile sites (24), and increased expression of oncogenes (25,26). Replication stress occurs when the replisome encounters such features on the DNA template, causing slowing or stalling of the fork, which, in turn, can lead to slower or reduced synthesis, fork collapse, DNA breaks, and checkpoint activation (27). The intra-S checkpoint allows for fork stabilisation and the prevention of origin firing, as well as the further slowing of DNA replication. Mutations in the checkpoint response proteins reveal the severe effects of prolonged replication stress. For example, mutations in the Ataxia Telangiectasia and Rad3-related (ATR) gene can cause Seckel syndrome (microcephalic primordial dwarfism), characterised by microcephaly and intellectual disability (28).

To avoid replication fork collapse or mutagenesis, and ultimately maintain genome stability, stalling impediments must either be resolved or bypassed efficiently. DNA repair mechanisms, such as nucleotide excision repair (NER), can be employed outside of S phase to remove damaged DNA nucleotides before the onset of replication. NER is a multistep process that involves several proteins (reviewed in (29)) and is particularly important for the removal of bulky lesions, like those introduced by ultra-violet (UV) light. Importantly, NER is a relatively slow process that is not infallible, and, additionally, lesions can arise during S phase. Therefore, unrepaired lesions are frequently present in DNA during replication, where they have the potential to affect polymerase progression.

The consequences of stalling events vary, depending upon which strand the arresting structure or lesion resides on. It is generally accepted that the constant cycles of priming during discontinuous synthesis reduces the impact of lagging strand lesions on fork progression, as a downstream primer can readily be synthesised as part of this canonical replication process. Providing the replicative helicase is not impaired by a lagging strand barrier, the lagging strand polymerase (Pol δ) can dissociate and restart replication from a new primer, bypassing the impediment (30). In fact, overall fork progression is hardly affected by lagging strand damage in reconstituted replisome collisions (31). The repair of stalling lesions can subsequently be conducted in a post-replicative manner. In contrast, large stretches of ssDNA are generated by leading strand polymerase stalling, caused by the continued unwinding of the DNA template by the replicative helicase; this is known as helicase-polymerase uncoupling (32). ssDNA is fragile and prone to breakage and is therefore protected by the binding of RPA. RPA binding acts as a marker of replication stress and can trigger the S phase checkpoint response by activating the ATR-mediated DNA damage response cascade. This prevents cell cycle progression when replication is incomplete (33). A wide variety of DNA damaging agents, including UV damage, crosslinking agents, polymerase inhibitors or stalling-induced replication stress, can localise and activate ATR by generating stretches of ssDNA. This is bound by RPA, and the 5′ primer end can be bound by the Rad9-Rad1-Hus1 (9-1-1) complex (34). This pathway, therefore, orchestrates multiple branches of the cell's replication stress response. The activity of ATR in the stress response pathway is reviewed in (35). ATR also decreases origin firing elsewhere in the genome, which prevents excessive ssDNA formation that would exhaust cellular RPA resources (36).

### Damage tolerance pathways: mechanisms to maintain active replication

**Figure 1.**
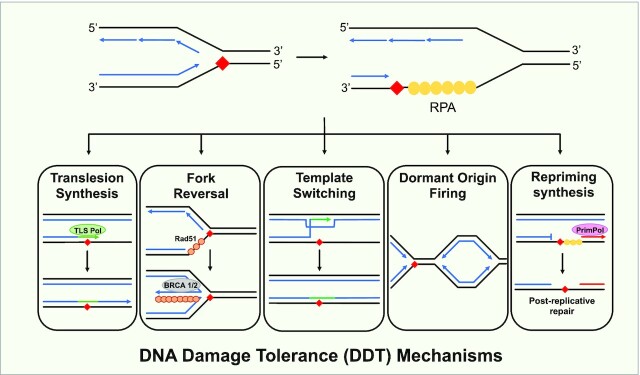
DNA damage tolerance pathways. Obstacles on the DNA template (red diamond) block ongoing DNA replication (blue arrows) and lead to fork stalling. This leads to helicase/polymerase uncoupling, generating tracts of ssDNA, which is bound by RPA (yellow circles). DNA damage tolerance mechanisms allow DNA replication to continue in the presence of such impediments. Translesion synthesis employs specialised polymerases (green oval) to insert bases opposite damaged templating bases (orange line indicates this insertion). Fork reversal begins as the recombinase Rad51 (orange circle) replaces RPA, and, along with the recruitment of additional factors, promotes the transient remodelling of a replication fork into a stabilised ‘chicken foot’ structure to allow for lesion repair or template switching. Rad51 and BRCA 1/2 (grey oval) are factors that prevent degradation of this reversed fork structure. Template switching requires strand invasion to use the newly replicated strand as a template instead of the damaged parental strand. Dormant origin firing is activated when the replication fork slows or stalls to ensure replication is completed in a timely manner. Dormant origin firing can occur alongside the other mechanisms of DDT. Finally, repriming requires *de novo* primer synthesis downstream of the lesion (red arrow) from which replication can be resumed by a replicative polymerase. In vertebrate cells, this is dependent on PrimPol (pink oval), which is recruited by RPA to ssDNA.

DNA damage tolerance (DDT) mechanisms are employed during S phase to bypass DNA lesions, structures and other obstacles without removing them and these impediments will be resolved by a variety of post-replicative pathways. This prevents fork stalling and allows DNA replication to continue in a timely manner, preventing replication stress. There are several mechanisms that cells rely on to continue replication past damage, including, translesion synthesis, template switching, fork reversal and firing of dormant origins (Figure 1).

Virtually all polymerases can perform synthesis across damaged DNA to some degree, but polymerases with high fidelity are the least adept at this process and are therefore prone to stalling. To tolerate damage, atypical bases can be bypassed by specialised polymerases during translesion synthesis (TLS) (37). These specialised Y family TLS polymerases (Pol k, Pol ι, Pol η, Rev1, and Pol ζ) can replace the replicative polymerase in an attempt to continue replication. TLS polymerases are able to accommodate distorted bases because they are endowed with more open active sites than the replicative polymerases. Because of this, TLS polymerases display low processivity, fidelity and efficiency, as their larger active sites interact less securely with DNA templates (38). Despite their inherent low fidelity, each specialised TLS polymerase is able to bypass at least one specific kind of DNA damage with relatively high fidelity: for example, Pol η accurately replicates over UV-induced cyclobutene pyrimidine dimers (CPD) lesions but is very inefficient at bypassing 6–4 photoproducts *in vitro* (39). Rev1 can bypass abasic sites by incorporating deoxycytidine bases (40). TLS polymerases lack the 3′-5′ exonuclease activity found in Pol δ and Pol ϵ, and this absence of ‘proofreading’ allows these polymerases to avoid enzymatic idling, where the proofreading exonuclease would remove any incorrect bases incorporated by the polymerase (41). The regulation of TLS polymerase activity is tightly controlled, in part by the activity of Proliferating Cell Nuclear Antigen (PCNA), a DNA clamp that forms part of the replisome. Monoubiquitination of PCNA by Rad6/Rad18 is a signal for the recruitment of TLS polymerases. However, polyubiquitination of PCNA—remarkably at the same amino acid, K164 (42)—will signal for the assembly of a different DDT pathway: template switching.

Template switching is a recombination-mediated mechanism of fork restart and is therefore significantly more accurate than using TLS polymerases, as the correct sequence can be copied from an undamaged template (43). The process of template switching involves the initial steps of TLS, including recruitment of Rad18 by RPA and chromatin remodelling by INO80. However, at the point of PCNA ubiquitination by Rad6/Rad18, Rad18 may recruit MMS2-UBC13 and HTLF/SHPRH, which polyubiquitinates K164 to stimulate template switching (44–47). The 9–1–1 clamp is then loaded to the 5′ end of the ssDNA, leading to Exo1 recruitment (48), and Rad51/BRCA2/Dss1 mediated strand invasion of the sister chromatid (49). This facilitates the synthesis of the unreplicated sequence opposite the damaged template by Pol δ. After replication has been completed, the newly synthesised strand switches back to its original position, leaving no unreplicated DNA but instead a sister chromatid junction (SJC) that requires resolution by BLM (Sgs1)/TOP3α (Top3)/RMI1/2 (RMI1) (50). This process is complex and requires the timely recruitment of a significant number of proteins, the formation and resolution of a D-loop and the resolution of an SJC before replication can continue. Unlike TLS, this process is considered to be error-free.

Fork reversal is another mechanism by which replication of a damaged template can be avoided, by using the newly synthesised nascent strand as a template. Fork reversal leads to the formation of a regressed fork, which is commonly referred to as a ‘chicken foot’ structure (51,52). This provides the cell with the opportunity to remove the DNA lesion after fork regression but before replication restart, or, alternatively, to bypass it through template switching once the fork restarts. Reversed forks can also converge with oncoming replication forks, bypassing the need for fork restart (53). Several factors have been implicated in protecting the reversed fork, including both BRCA1 and BRCA2, and the binding of Rad51 to RPA covered ssDNA. Fork reversal is dependent on the action of SMARCAL1, HLTF or ZRANB3 (54). The majority of fork reversal mechanisms have only recently been reported (reviewed in (55)) and further studies are required to fully elucidate the molecular mechanisms underpinning this process.

An additional method employed by cells to tolerate replication stress is dormant origin firing, a mechanism by which the inactive origins distributed throughout the genome are activated. In G1, when the MCM complex is loaded onto origins, significantly more origins are loaded with inactive complexes than are initially activated. The remaining inactivated origins can then be activated in response to replication stress, despite the activation of the ATR-dependent S phase checkpoint, which decreases late-stage origin firing (56). In fact, Chk1, required for the suppression of origin firing, is paradoxically required for the dormant origin activation by distinguishing between origins within currently active replication factories and those outside (57).

While all of these DDT pathways are now well established, the existence of another conserved mechanism for the bypass of replication fork barriers has been debated by the field for over half a century. The canonical model for discontinuous lagging strand synthesis has long been accepted and, in keeping with this, lesion bypass can be explained simply by constant cycles of priming. However, the existence of a bespoke pathway to reprime stalled leading strand synthesis has been the subject of much debate. Here, we review the available evidence for repriming as a canonical mechanism that promotes DNA damage tolerance and replication restart during leading strand duplication.

### Early investigations to elucidate a model of DNA replication

In the years following the discovery of the structure of double-stranded DNA (dsDNA) by Watson and Crick, the field moved quickly to develop a model that described the mechanism of its duplication (58). The isolation of the first DNA polymerase (*Escherichia coli* DNA polymerase I) in the late 1950s provided the first example of an enzyme with the ability to catalyze the synthesis of new DNA strands (59). Interestingly, this polymerase synthesised DNA in a specific 5′ to 3′ direction, which has since been shown to be an inherent feature of all known polymerase enzymes (60). This directionality of synthesis posed an interesting question regarding the nature of replication of each of the anti-parallel strands in dsDNA. While one strand could, theoretically, be replicated continuously as the DNA is unwound, the other strand must somehow be replicated backwards (3′ to 5′) to allow for coupled, unidirectional fork progression.

In a classic study investigating replication intermediates in *E. coli*, Okazaki *et al.* used alkaline sucrose gradient sedimentation approaches to uncover low molecular weight (LMW) DNA fragments (Okazaki fragments), synthesised during a quick pulse of radioactive labelling (61,62). The failure to detect any high molecular weight (HMW) molecules after short pulse times led to the postulation that all DNA is synthesised in small pieces. By adding a chase of unlabelled nucleotides into the protocol, the conversion of the radioactive LMW intermediates into fragments of HMW could be observed, hinting at the existence of a joining process and confirming that the small fragments observed were, in fact, intermediates of chromosomal DNA synthesis (61). Subsequent studies found that the newly synthesised DNA fragments were assembled into larger molecules by the further joining of additional fragments to the 3′ end of pre-existing material, as would be expected (63,64). The DNA ligase enzyme was later implicated in the joining of the small fragments, and, accordingly, almost all DNA is present in small molecules in cells expressing temperature-sensitive ligase mutants at non-permissive temperatures (65,66). DNA ligase was also shown to join these fragments in both *Saccharomyces cerevisiae* and *Schizosaccharomyces pombe* (67,68), and human DNA ligase I is now well characterised in this role (reviewed in (69)). *E. coli* harbouring mutations in DNA polymerase I (PolA) also displayed an impairment in the joining of LMW fragments into HMW molecules (70). This suggested a model where PolA fills in gaps between fragments before DNA ligase catalyses the formation of a phosphodiester bond to seal the individual pieces together.

Okazaki's findings offered a solution to the directionality problem, whereby the strand requiring synthesis in the 3′ to 5′ direction (now known as the lagging strand) could be synthesised in short, discontinuous fragments, which can subsequently be ligated into a completed product. Supporting this, the small fragments isolated by Okazaki *et al.* were shown to contain short stretches of RNA, which provided insights into the mechanism by which they are produced (71). Since DNA polymerases are incapable of *de novo* synthesis, short RNA primers would be required at frequent intervals on the exposed lagging strand to act as substrates for the initiation of DNA synthesis by the replicative polymerase. The presence of these RNA species signified that the short fragments are the result of true initiation events and established a model for lagging strand synthesis that allows replication to progress in the same direction as its anti-parallel partner strand, which is also replicated in a 5′ to 3′ direction (71).

**Figure 2.**
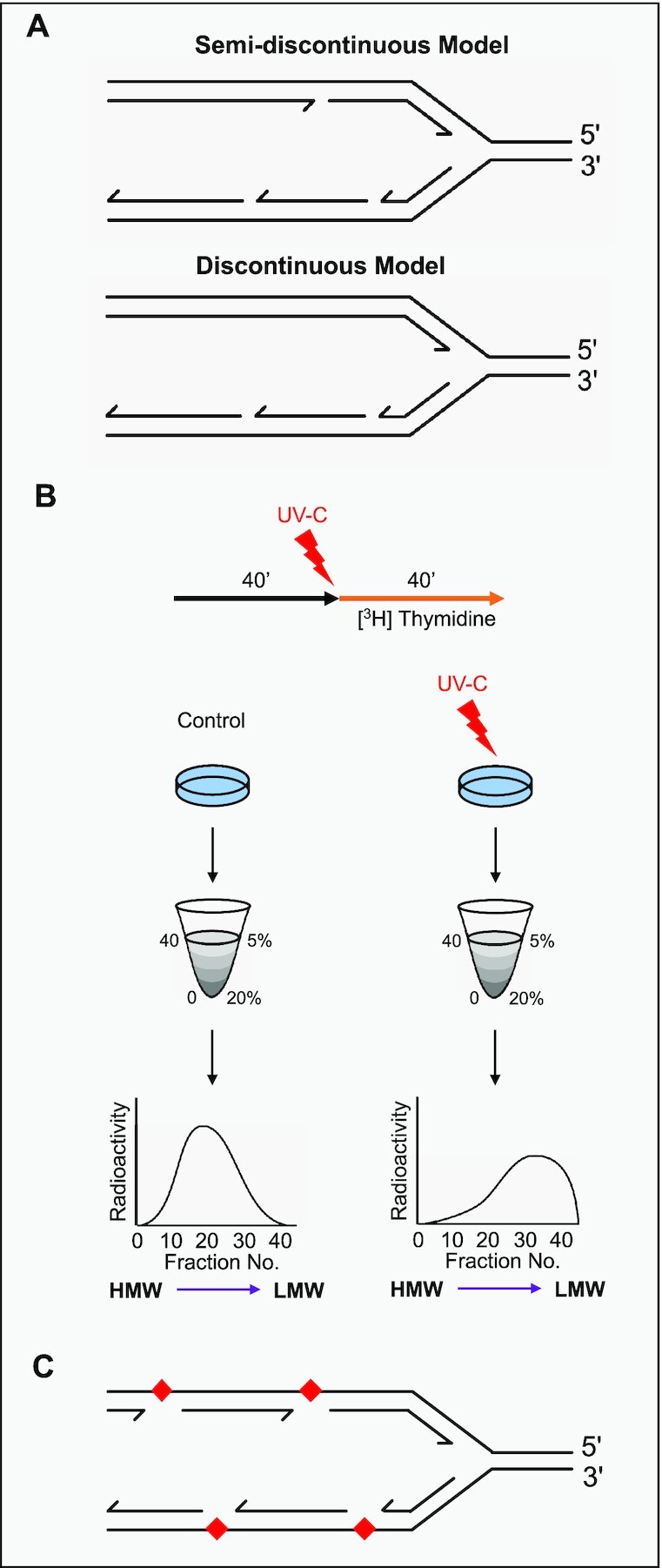
Uncovering models of DNA replication. (**A**) In the semi-discontinuous model of DNA replication, leading strand synthesis is continuous from origin to termination and the lagging strand is synthesised in short fragments. Theoretically, if ligation is prevented, two size classes of replication intermediates would be produced: a HMW continuous leading strand and LMW fragments from the lagging strand. In the discontinuous model, both strands of DNA are synthesised as fragments and all DNA initially consists of LMW fragments. (**B**) The protocol used in the seminal studies of Rupp and Howard–Flanders to investigate DNA replication intermediates in bacteria following UV damage. *Escherichia coli* cells were grown in unlabelled medium (black arrow) before being irradiated with UV-C and transferred to media containing radioactive thymidine (orange arrow). After 40 min of labeling, the cells were collected for analysis. DNA was harvested from either control or irradiated cultures and subject to alkaline sucrose gradient centrifugation. The sedimentation showed that DNA fragments extracted from irradiated cells were significantly smaller than those from control cells. (**C**) The results were interpreted to indicate that gaps were present in the nascent DNA opposite the CPDs (red) induced by UV irradiation.

Following these seminal studies, there was considerable debate regarding the nature of leading strand (5′ to 3′) synthesis; was it synthesised continuously from the origin to termination (semi-discontinuous model) or did synthesis frequently start and stop in a similar manner to the lagging strand (discontinuous model) (Figure 2A)? Logically, replication restart on the leading strand seems unnecessary, as continuous 5′ to 3′ synthesis from the origin until termination is mechanistically possible. In theory, the 3′-OH of the nascent leading strand can prime further replication and, in addition, discontinuous synthesis would be more energy- and time-consuming. However, all replication intermediates detected in Okazaki's studies were LMW fragments, supporting a discontinuous model where synthesis is reinitiated frequently on both strands. The evidence put forward to settle this debate over the coming decades was conflicting.

Despite the early evidence pointing towards a fully discontinuous model of replication, later studies introduced contradictory evidence that supported the simpler semi-discontinuous model which, perhaps due to its practical appeal, was well received by the field. The results of studies utilising an *in vitro E. coli* DNA synthesis system provided compelling evidence in support of continuous leading strand synthesis (72). Fragments of DNA produced in this system during ligase inhibition were reproducibly shown to fit into one of two distinct size classes; Okazaki fragments that were produced with a low sedimentation coefficient and a distinct class of larger labelled molecules. Interestingly, the distribution of DNA between the two classes was roughly equal and further investigation confirmed that fragments in one class were complementary to fragments in the other, a sign that they originated from opposing (leading or lagging) strands (73). These data were indicative of one strand being synthesised continuously while the other one was produced discontinuously, adding support to the semi-discontinuous model. In addition, multiple studies exploiting rolling circle-type DNA replication systems were able to produce long leading strand products of 40–500 kb in length, with no evidence of dissociation (74,75). Thus, the semi-discontinuous model of replication was well supported by *in vitro* studies (72–75).

The evidence regarding the nature of bacterial leading strand synthesis was often contradictory between the published *in vitro* and *in vivo* studies, with the latter usually supporting a fully discontinuous model. However, evidence supporting the use of a continuous mechanism of leading strand synthesis *in vivo* is provided in some early literature. Iyer and Lark investigated the mechanism of production of intermediate molecular weight and HMW replication intermediates that were generated during pulse labelling experiments (63). Their results showed nucleotides being added to the 3′ end of nascent DNA strands, suggesting continuous synthesis. Direct *in vivo* evidence for a semi-discontinuous model was later reported, however, this was dependent on the presence of PolA, which is now known to fill in gaps generated by discontinuous synthesis, as described earlier (76). The method of reaction termination (pyridine-KCN) used in the two studies presented above has since been called into question (77). The use of a pyridine-KCN termination pulse permits the ligation of nascent DNA fragments after application, and this is likely the source of the long ‘continuous’ fragments. By using a more rapid and robust method of termination, it was shown that all nascent DNA fragments were short *in vivo*, agreeing with previous studies and supporting a discontinuous model of replication (77).

Subsequent studies set out to explain the disparities between the *in vitro* results and those observed in biological systems. One possibility that had not been excluded by early studies was that the fragments observed *in vivo* could be a result of DNA processing or excision repair activities. Uracil is a common lesion present in DNA, resulting from either deamination of cytosine or misincorporation of deoxyuridine 5′-triphosphate nucleotide (dUTP). In order to maintain genomic integrity, uracil must be detected and removed by base excision repair (BER), a process that generates breaks, or gaps, in the backbone of the DNA chain. Examining DNA synthesis in *E. coli* lysates had previously uncovered two size classes of intermediates (72). Increasing the concentration of dUTP present in the lysate solution led to a decrease in the sedimentation coefficient of the larger size class, representing the generation of smaller fragments (78). The addition of dUTP to *in vitro* reactions produced smaller DNA fragments with a sedimentation profile that was similar to DNA obtained from *in vivo* experiments. The conclusion was, therefore, that the small DNA fragments observed in previous experiments could be explained simply by dUTP incorporation and excision and it was deemed no longer necessary to consider possible leading strand reinitiation events. However, this conclusion was strongly refuted by other evidence published at the same time (79). Comparing the sedimentation profiles of DNA produced by ligase-deficient *E. coli* to DNA from a strain that was also deficient in excision-repair of uracil demonstrated little or no difference in the sizes of fragments produced by either strain *in vivo*. Subsequent studies also concluded that neither DNA processing nor uracil excision were found to affect the size of replication intermediates (80). While this added support to the idea of multiple initiation events on both strands, it was not direct evidence and the semi-discontinuous model was generally still considered to be the most convincing.

A recent study has revisited the questions surrounding the origin of replication intermediates in bacteria (81). Surprisingly, nearly all of the LMW leading strand products observed in earlier studies can be explained by fragmentation as part of excision-repair processes. Mutants deficient in BER, mismatch repair (MMR), NER and ribonucleotide excision repair (RER) were able to perform largely continuous synthesis on the leading strand, suggesting that these could be responsible for fragmenting DNA. In particular, the RER pathway is responsible for most fragmentation events, which could explain why earlier studies investigating dUTP excision failed to detect a noticeable effect (79,80). The implication of this work is, therefore, that all DNA is initially synthesised with a number of incorrect bases and requires extensive excision repair in order to become mature DNA. Such events generate discontinuities in the nascent chain that produce the DNA fragments detected in previous studies. Interestingly, their data did not show chromosome-length continuous fragments in the absence of any excision pathways, in fact, DNA fragmented in two size classes, with the largest class of fragments determined to be 50–70 kb. This supports Okazaki's original model for the discontinuous synthesis of both strands of DNA, albeit with two size classes of fragments, presumably originating independently from each of the DNA strands.

### Studying replication after damage: new insights into the replication model

Although the studies described above helped to delineate a working model for the canonical mechanism of DNA replication in unperturbed conditions with normal amounts of fork stalling caused by endogenous sources, examining how DNA is copied following the application of fork stalling agents also provided critical insights into how this duplication process operates. In the late 1960s, Rupp and Howard-Flanders conducted a seminal study, which explored the fate of DNA when cells were permitted to replicate following UV damage (82). By utilising NER-deficient *E. coli* strains, UV-induced pyrimidine dimers could persist into S phase. At the time, it was unknown whether replication would be stalled by these photo-lesions or continue past this damage with minimal perturbation. By measuring tritiated thymidine incorporation following UV irradiation, they determined that each lesion caused ∼10-s delay to the replication fork, however, the lesions did not completely block replication. This observation prompted the central question of the study: was the bypass of UV lesions continuous or discontinuous in nature? To address this question, they utilised rapid pulses of radioactive labelling to mark newly synthesised DNA in damaged and undamaged cells that could be subjected to alkaline sucrose gradient centrifugation for comparison (Figure 2B). DNA originating from cultures that weren’t exposed to UV sedimented in large pieces, however, strikingly, DNA synthesised following UV exposure sedimented in significantly smaller pieces; a sign of discontinuities in the nascent chains. The results were interpreted to indicate the presence of single-stranded gaps in the DNA of daughter strands following UV exposure. Interestingly, the gaps observed were spaced at distances roughly correlating to the predicted distance between CPDs produced at the specific UV dose used, suggesting the gaps may reside opposite damaged bases (Figure 2C). Further work demonstrated that these ssDNA gaps were ∼1000–2000 bp in length (83). Over time, the ssDNA gaps opposite CPDs were repaired by sister-chromatid exchange (SCE) to produce detectable full-length chromosomal DNA (84). The discovery of gaps in all nascent DNA following damage led to the postulation that replication restart downstream of polymerase-stalling damage could occur on both strands of DNA.

Repeating the initial experiments conducted by Rupp and Howard-Flanders in mammalian (Chinese hamster) cells produced ssDNA gaps similar to those observed in *E. coli*, which were also filled in over time (85). Gaps were later discovered in DNA from human cells following UV irradiation (86). In contrast to the results in *E. coli*, no evidence of gap-filling by an SCE mechanism could be found in mammalian cells; however, there was evidence of gap filling by DNA synthesis that was not coupled to SCE (87). The evidence for discontinuous synthesis after damage provided by these early studies suggested an inherent ability of the replisome to skip synthesis opposite a lesion or replicative impediment and restart replication downstream on both the leading and lagging strand. One model proposed to explain the observed results involved the generation of a *de novo* primer downstream of a stalling lesion, from which the replicative polymerase can resume synthesis, as occurs on the lagging strand.

Following the publication of these studies, there was little further work into resolving the questions surrounding discontinuous leading strand synthesis. Replication restart downstream of lesions was still considered unlikely and the leading strand was generally considered by the field to be synthesised continuously (88). The discovery of TLS polymerases in the late 1990s provided a compelling solution for lesion bypass that didn’t require reinitiating synthesis on the leading strand (89–91). The ability of these enzymes to synthesise past damaged bases allowed the development of new models, which almost all involved polymerase switching to a TLS enzyme at the active fork, maintaining continuous synthesis of the nascent chain. The solutions offered by models involving the newly discovered TLS pathways were preferable to models which went against the dogma of continuous leading strand synthesis.

In the early 2000s, the debate over continuous versus discontinuous leading strand synthesis was still ongoing (92), then, in 2006, two significant studies provided compelling evidence supporting a model where leading strand synthesis can be initiated downstream of a lesion, prompting a re-evaluation of the semi-discontinuous model. The first study combined 2D gel electrophoresis with electron microscopy to inspect DNA derived from UV-irradiated NER-deficient *S. cerevisiae* cells (93). Single-stranded DNA gaps in both strands were directly visualised behind the replication fork. In WT cells, the gaps were filled in over time. However, in cells deficient in TLS or homologous recombination, gaps persisted after completion of S phase suggesting they are repaired by a post-replicative repair mechanism(s). The second study provided the first mechanistic evidence supporting the existence of a repriming mechanism in *E. coli*, as Rupp and Howard-Flanders had originally proposed (94). By using a terminal 3′ dideoxynucleotide on the simulated nascent leading strand of a forked template, Heller & Marians showed that synthesis could resume downstream of the blocked end, without repair of the lesion; a process that would require *de novo* synthesis (94). This discontinuous synthesis was dependent on both the replicative primase (DnaG) and helicase (DnaB), suggesting that primer synthesis can take place on the leading strand to allow replication to resume after fork stalling events.

### Roles of replicative primase enzymes in leading strand replication

As discussed above, both leading and lagging strand priming is performed by the replicative primase DnaG in *E. coli* (94,95). The roles of DnaG and DnaB (helicase) in repriming replication restart have now been also established (96,97). It is, however, important to note that although bacterial replisomes bear many mechanistic similarities to that of eukaryotes, both systems have seemingly evolved independently (reviewed in (98)). One key difference in their mechanisms is the direction of travel of their respective replicative helicases. In *E. coli*, DnaB traverses along the lagging strand in a 5′ to 3′ direction, while the eukaryotic MCM helicase moves 3′ to 5′, placing it on the leading strand (99,100). Furthermore, as discussed previously, the eukaryotic system divides the labour of bulk synthesis between Pol ϵ and Pol δ, whereas the majority of *E. coli* replication is conducted by multiple copies of the C-family polymerase, DNA Polymerase III (101). Interestingly, the primase enzymes of each domain of life are also distinct, despite their functional similarities. Bacterial DnaG primases are more closely related to topoisomerases, both having a common TOPRIM fold (102). In contrast, the so-called eukaryotic primases (Pri1/PriS) evolved from a primordial RNA recognition motif (RRM) with a diverse range of distantly related homologues found in all domains of life, albeit their specific roles in priming genome replication appears to be restricted to viruses, archaea and eukarya (103).

During eukaryotic replication, RNA-DNA primers are generated by the Pol α-primase complex, consisting of four distinct subunits: p180, p74, p58 and p48 (104). The primase is formed of the latter two subunits, with p48 (Pri1/PriS) acting as the catalytic subunit and p58 (Pri2/PriL) acting to stabilise the primase (105). A temperature-sensitive mutant of the budding yeast Pri1 subunit has allowed investigation of the role of the primase *in vivo* (106). DNA synthesis in the mutant is partially defective at the permissive temperature, however, at the restrictive temperature, DNA synthesis fails at an early step following release from G1 arrest. These results indicated that the primase is required to maintain ongoing DNA synthesis. Interestingly, cells expressing the mutant primase fail to slow the rate of S phase progression following methyl methanesulfonate (MMS)-induced DNA damage. Investigating this effect further revealed evidence suggesting a role for Pri1 in the Rad53p-dependent checkpoint pathways that regulate cell cycle progression in response to DNA damage. Pri1 mutants have recently been shown to experience an increase in premature sister chromatid separation (107).

Reconstituting the yeast replisome *in vitro* has facilitated further studies into the roles of Pol α-primase and provided insights into priming events on the leading strand. In one study, the Pol α-primase complex was shown to synthesise a primer on the leading strand of a forked substrate and then extend this using its polymerase activity, or hand over to Pol ϵ or Pol δ (108). Additionally, when a primer was provided, the enzyme complex preferentially extended this, rather than synthesise a *de novo* primer. The study found no evidence of repriming on the leading strand. In another recent study, lagging strand priming by Pol α-primase complex to bypass a CPD was found to be fast and efficient, while leading strand repriming was inefficient for re-establishing replication beyond the lesion (31). Interestingly, the efficiency of leading strand repriming was related to the availability of RPA, where depleting the pools of RPA increased priming efficiency. Pol α-primase has long been thought to prime the leading strand at the origins. However, a recent examination of the establishment of bi-directional leading strand synthesis in a reconstituted yeast system determined that leading strand synthesis is, in fact, initiated from a lagging strand primer on the opposite side of the origin (109). Overall, this evidence suggests that budding yeast Pol α-primase does not play a major role in priming leading strand synthesis, at least at origins, nor does it appear to efficiently reprime on this strand to promote damage tolerance.

While the availability of temperature-sensitive mutants and *in vitro* reconstituted replisome systems have allowed the study of some of the functions of the budding yeast primase, the mammalian enzyme has proven more difficult to study. In a similar manner to the yeast homologue, human Pol α-primase forms the replicative primase complex that is composed of the DNA polymerase α subunits (POLA1 and POLA2) and the DNA primase subunits PRIM1 and PRIM2 (110). There is currently no substantial evidence to suggest that the human Pol α-primase complex plays a role in DNA damage tolerance on the leading strand. Therefore, in contrast to prokaryotic cells, it is unlikely that stalled leading strand synthesis in eukaryotic cells can be restarted from downstream primers synthesised by the replicative primase.

The observations described above raise important questions regarding the strand-specific differences in priming efficiency displayed by Pol α-primase. What enables the enzyme to regularly prime the lagging strand efficiently, while priming on the leading strand is so inefficient? Most ssDNA is rapidly coated in RPA, which has been shown to inhibit Pol α-primase activity, however, despite this, the lagging strand is primed efficiently by this complex (31). During replisome progression, the CMG encircles and translocates along the leading strand, while the lagging strand is excluded (6). Pol α-primase is kept in close proximity to CMG via an interaction with Ctf4 (AND-1 in humans), and it is possible to consider that this proximity may prevent RPA from binding the lagging strand before it reaches the primase, allowing efficient priming to take place during Okazaki fragment generation (111). Outside of this specific scenario, for example during helicase uncoupling after leading strand stalling events, ssDNA is rapidly coated in RPA, which would prevent Pol α-primase from priming or repriming (31). Another possibility is that Pol α-primase could be regulated by auxiliary factors that limit its usage. For example, while the absence of Ctf4 in reconstituted yeast systems does not seem to affect lagging strand synthesis on chromatin *in vitro*, Ctf4 has been suggested to aid the maintenance of robust lagging strand priming when Pol α-primase activity is reduced *in vivo* (112,113). The human Ctf4-orthologue, AND-1, interacts with Pol α-primase via its C-terminal HMG box and displays DNA-binding activity, potentially providing a mechanism by which Pol α-primase is directed to the lagging strand (114).

### Discovery of a new class of eukaryotic primase

In the past, DNA primase enzymes were thought to possess one specific function: synthesising short RNA primers during the initiation of DNA replication. However, more recently, this has been shown to be somewhat of a functional mis-annotation and nowhere is this more evident than in members of the archaeo-eukaryotic primase (AEP) superfamily (reviewed in (103)). Enzymes belonging to this superfamily can be found throughout all domains of life, where they have evolved specialist roles in replication, repair and DNA damage tolerance.

Perhaps the best-known family member is Pri1 (PriS) which, in complex with the large subunit (Pri2/PriL), synthesises RNA primers during canonical origin firing and lagging strand synthesis (13). In archaea, Pri1 can extend primers with dNTPs in a manner similar to Pol α, which is lacking from these organisms (115). Archaeal Pri1 can also conduct TLS over various helix-distorting lesions to facilitate DNA damage tolerance (116). Pri1 is not the only AEP discovered in archaea; for example, the archaeal cryptic plasmid pRN1 encodes an enzyme, ORF904, which contains a helicase/translocase domain in addition to the AEP domain that renders the protein proficient in both primase and polymerase activities (117). In fact, ORF904 can synthesise many kilobases of DNA when conducting bulk replication of pRN1 plasmids. In addition, many bacterial species possess various AEP orthologues. For example, RepB’ and Rep are AEPs found on RSF1010 and ColE2 plasmids, respectively, that more conventionally generate short primers to initiate plasmid replication (118,119). Perhaps even more intriguing is the discovery of AEP proteins that are co-operonic with bacterial non-homologous end joining (NHEJ) protein Ku (120,121). Here, the AEP protein forms part of a larger DNA break repair complex known as Ligase D (LigD), which further associates with Ku (Ku-LigD complex) to facilitate prokaryotic NHEJ (122). In mycobacteria, Prim-PolC is co-operonic with Ligase C and plays a role in excision repair (123), binding to the short gaps produced as part of this excision process and conducting gap-repair synthesis (124). The AEP family has recently been renamed as Primase-Polymerases (Prim-Pols) to better reflect the more diverse origins and functions of this replicase superfamily (103).

In 2005, a bioinformatic study identified a variety of novel Prim-Pols, including a second Prim-Pol gene in the human genome called CCDC111 (125). This gene product was subsequently isolated and characterised (126–128). The protein was shown to be a DNA-dependent DNA polymerase that also possesses TLS-like activities on lesion-containing templates, such as 8-oxo-G and 6–4 pyrimidine dimers. In addition, the enzyme showed robust primase activity on DNA templates. However, in contrast to replicative primases, it utilises dNTPs much more efficiently than rNTPs (129). To reflect both of these capabilities, CCDC111 was renamed Primase-Polymerase (PrimPol). Human PrimPol is a monomeric enzyme (130), differing from replicative primase enzymes, which form heterodimers, such as the eukaryotic primase complex Pri1/Pri2 (131). PrimPol contains a characteristic N-terminal AEP domain containing three conserved motifs (I, II and III) that are essential for all catalytic activities. Motif I contains residues (DxE) that create a binding site for divalent metals (125) and mutating these residues ablates catalytic activity (126–128). A UL52-like zinc finger (ZnF) domain is located downstream of the catalytic domain. This domain contains a conserved sequence (Cys-His-Cys-Cys) that allows the coordination of a metal ion to form a zinc finger. The ZnF domain binds to ssDNA and appears to play a role in PrimPol's priming mechanism, as mutating/deleting it abolishes primase, but not polymerase, activity (130,132,133). The C-terminal domain (CTD) of PrimPol binds to the single-strand binding protein, replication protein A (RPA70) (128,134) and PrimPol foci formation is dependent on this interaction (135). A recent study elucidated the molecular basis of this interaction and identified two RPA binding motifs, RBM-A and RBM-B, contained within the CTD, which interact with the basic cleft of RPA70N (135). PrimPol also binds to PolDIP2 (PDIP38) and this enhances its polymerase, but not its priming, activities (136), although the specific cellular role of this complex remains to be established.

### Establishing a role for PrimPol in vertebrate cells

Orthologues of PrimPol are found in most eukaryotic organisms, with a few notable exceptions, such as *S. cerevisiae, S. pombe, Caenorhabditis elegans* and *Drosophila melanogaster*. Our current understanding of the enzyme's *in vivo* functions comes predominantly from avian and human cell studies. PrimPol knockout avian cells (DT40) show a pronounced sensitivity to UV-C damage, 4NQO (a UV mimetic), cisplatin, chain-terminating nucleotide analogues (CTNAs) and MMS, but no greater sensitivity to agents that induce double-strand breaks (126,137,138). These cells also exhibited a distinct G2-M checkpoint response after UV damage (137). In contrast to Pol η knock out cells, which also display UV-C sensitivity, no loss in post-replicative repair of UV-C damage was observed when PrimPol is depleted (130,137). Fork speeds and general fork progression are decreased in PrimPol's absence. This is especially prominent following UV damage, strongly suggesting a role for PrimPol in the maintenance of fork progression after DNA damage.

The importance of PrimPol in DNA damage tolerance was further supported by studies of human PrimPol^−/−^ MRC-5 cells (139), which also showed decreased fork speeds and increased fork stalling after damage, although the damage sensitivity observed in avian cells was not observed in the human knock out (or knock down) cells (126). This discrepancy is likely due to the significantly shorter doubling time of DT40 cells compared to human cells – 11 hours compared to 24 hours (140,141). Human PrimPol^−/−^ cells also exhibit a variety of phenotypes that highlight the important roles this protein plays in maintaining DNA stability in both the nucleus and mitochondria. These include increased micronuclei, sister chromatin exchanges and mutation frequency (139).

**Figure 3.**
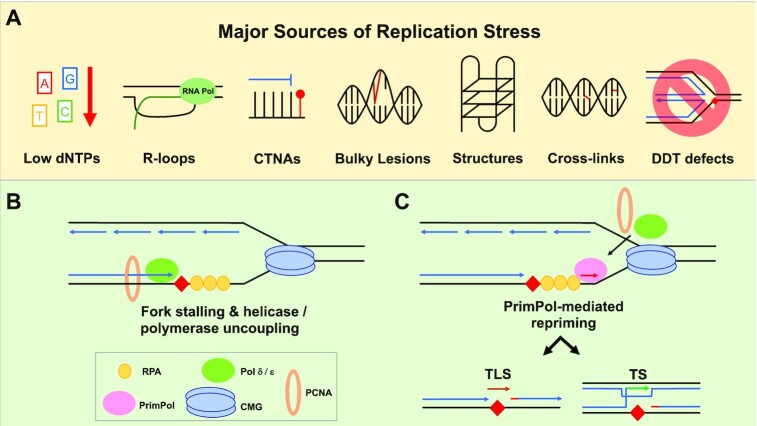
PrimPol-dependent repriming of stalled replication intermediates. (**A**) PrimPol-mediated repriming can assist in resolving fork stalling after many different kinds of lesions, including CTNAs, bulky lesions such as those generated by UV light, G-4 quadruplexes, R-loops, and intra/interstrand crosslinks. PrimPol can also be utilised when low dNTPs pools cause fork stalling. Additionally, the absence or loss of an alternative DDT pathway, such as fork reversal, can lead to the deployment of a PrimPol-dependent pathway. (**B**) Replication fork uncoupling occurs when lesions, or other sources of replication stress, transiently stall the replicative polymerase without impeding the rest of the replisome. This uncoupling generates stretches of ssDNA onto which RPA can bind. (**C**) PrimPol (pink oval) can be recruited to these tracts of RPA bound ssDNA to facilitate the restart of the uncoupled fork by repriming. From here, the replicative polymerase will take over to complete synthesis. The repriming depicted here occurs on the leading strand, with lagging strand machinery omitted for clarity.

While PrimPol evidently plays an important role in tolerating lesions, it is important to acknowledge that PrimPol is also involved in maintaining replication during stress (Figure 3A). Hydroxyurea (HU) slows and stalls replication by inhibiting ribonucleotide reductase, thus depleting the cellular dNTP pool. Upon treatment with HU, human cells exhibit both an increase in chromatin-bound PrimPol and a relocalisation of PrimPol into subnuclear foci (126,132). In PrimPol depleted cells, HU treatment causes a decrease in fork progression, as measured by DNA fibre analysis, which can be rescued by expressing wild-type PrimPol, but not a primase-deficient version of the enzyme (132). A recent CRISPR screen implicated PrimPol in the response to resveratrol and its chemical analogue pterostilbene (142). Like HU treatment, Resveratrol also induces comparable dNTP depletion and fork speed decrease, highlighting the drug's ability to cause replication stress. Overall, these studies suggest that PrimPol also performs a more general ‘house-keeping’ role in maintaining unperturbed fork progression in response to replication slowing and endogenous fork stalling.

### PrimPol reprimes downstream of lesions and stalling structures *in vivo*

Recent studies have begun to establish the roles that PrimPol plays *in vivo*, which ultimately underlie the phenotypes observed in its absence. Early studies noted that PrimPol was required to maintain replication fork speed following UV exposure and that this effect was dependent on its primase activity (Figure 3A) (126,130,132). Interestingly, previous studies had already suggested that UV-stalled forks were restarted via repriming in human cells (87,143). There is also evidence that PrimPol reprimes downstream of AP-sites, and this activity has been suggested to allow some cells to tolerate the mutagenic lesions produced by the APOBEC/AID family of cytosine deaminases (144). Using avian cells, it was shown that PrimPol also mediates tolerance to chain-terminating nucleoside analogues (CTNAs) (Figure 3A), which stall DNA replication (138). Cells deficient in PrimPol experienced a significant decrease in survival after treatment with CTNAs, which can be complemented by the introduction of WT PrimPol, but not a primase-deficient mutant. The mechanism underpinning the tolerance was supported by *in vitro* studies demonstrating that PrimPol could synthesise a *de novo* primer ∼14 nucleotides downstream of a CTNA present at the 3′ end of a primer strand (138). A depiction of replication restart mediated by PrimPol following fork stalling is shown in Figure 3B and Figure 3C.

PrimPol has additionally been implicated in the tolerance of DNA structures, such as G4-quadruplexes (Figure 3A) (145). Using histone recycling as a measurement of replisome uncoupling in DT40 cells, Schiavone *et al.* found local epigenetic instability in the absence of PrimPol around the *BU-1A* locus when a G4 structure was present on the leading strand. While PrimPol does not directly replicate G4′s, it was shown to reprime downstream of these structures, allowing rapid resumption of replication and preventing replisome uncoupling. The system was later adapted to study the potential role of PrimPol in R-loop bypass by replacing the G4 quadruplex sequence with R-loop forming purine-rich repeats of (GAA)_*n*_ (146). In a WT background, short tracts of repeats (*n* = 10) did not affect the epigenetic stability, indicating that the replisome can move through the region unhindered. Strikingly, in PrimPol knock-out cells, the same short tracts caused a significant increase in the local epigenetic instability, indicative of fork stalling, which could only be rescued by expression of primase-proficient PrimPol.

The abilities of PrimPol demonstrated by the studies described above highlight its role in the tolerance of a myriad of fork-stalling lesions and structures (Figure 3A). Interestingly, while multiple studies of PrimPol have demonstrated TLS capabilities *in vitro*, evidence supporting its use *in vivo* remains to be established. Hence, the current consensus is that PrimPol's primary role *in vivo* is to reprime DNA synthesis; while a role in TLS cannot be ruled out, it can be assumed that the majority of phenotypes observed in the absence of PrimPol are caused by the cell's inability to reprime stalled DNA replication, particularly on the leading strand (126,127,130,132). Previously, there was no known mechanism to facilitate repriming on the leading strand in vertebrate cells; however, considering all of the evidence now available, it is apparent that PrimPol-mediated repriming provides cells with a highly flexible mechanism for restarting DNA synthesis and bypassing obstacles on the leading strand thus preventing replication stress. It should be noted that PrimPol may also prime on the lagging strand, however, this would seem redundant given the activity of Pol α-primase. The discovery and characterisation of a PrimPol-dependent repriming pathway also provides additional evidence to support a model for discontinuous synthesis on both strands during DNA synthesis.

### Repriming DNA synthesis represents a canonical damage tolerance pathway

While evidence for the existence of DNA damage tolerance mechanisms that involve repriming downstream of replicase stalling obstacles has been invoked for over half a century, it is still rarely regarded as an actual canonical DDT pathway. This might be because the replicative primases of prokaryotes, and probably some other organisms too, have the intrinsic ability to reprime DNA synthesis and are, therefore, not considered to represent a distinct DDT pathway. Furthermore, the roles of other Prim-Pol enzymes in various other genome stability pathways have only recently been appreciated (103). In addition, the relatively mild phenotypes displayed by human cells depleted of PrimPol do not, at an initial glance, mark this out as a major pathway of DDT. While PrimPol deficient human cells do exhibit a change in cell cycle profile and modest slowing of replication forks, they do not display overt signs of distress or growth impediments (139). Interestingly, the effects of losing PrimPol seems to vary between organisms. For example, avian PrimPol^−/−^ cells exhibit more pronounced phenotypes, specifically a sensitivity to fork stalling lesions, in addition to profound G2 stalling after UV damage that is only partially resolved by the application of Chk1 or p38 inhibitors (126,137). PrimPol^−/−^ mice remain viable without displaying any overt phenotypes. Mouse embryonic fibroblasts (MEFs) lacking PrimPol display increased chromatid breaks, suggesting the generation of lesions during S phase (126,127). Depletion of a PrimPol orthologue, PPL2, in trypanosomes results in a lethal mitotic catastrophe-like phenotype, likely due to replication defects, highlighting an essential role for PrimPol in these protists (147).

An ever-increasing number of recent studies are reporting functional overlaps between repriming and other DDT pathways, suggesting that the impact of repriming is often underestimated, as its absence can be compensated for by other pathways. The first such overlap to be described was observed while studying PrimPol depletion in xeroderma pigmentosum variant (XP-V) cells (126,139). XP-V cells contain mutations in the *POLH* gene, which encodes the TLS polymerase Eta (Pol η) (91). Pol η provides tolerance to UV-induced damage by conducting error-free bypass of CPD lesions. Due to PrimPol's proficiency in conducting TLS-like bypass of UV-induced lesions (6–4 PPs) and extending from CPDs *in vitro*, it was hypothesised that the two enzymes could work in complementary pathways (126). To test this, PrimPol was depleted in both WT and XP-V cells before applying a dose of UV radiation. Both cell types exhibited increased RPA foci and a concurrent increase in phosphorylation of the intra-S checkpoint kinase Chk1 in response to UV-induced damage. Interestingly, in cells lacking both Pol η and PrimPol, levels of Chk1 phosphorylation remained elevated for significantly longer than cells deficient in only one enzyme, suggesting a complete deficiency in UV damage bypass when both enzymes are removed. While both PrimPol-depleted fibroblasts and XP-V exhibited either absent or mild UV-C sensitivity, in the absence of caffeine, PrimPol-depleted XP-V cells become synergistically sensitive to UV irradiation, establishing a non-epistatic relationship between these distinct DDT pathways. PrimPol's role in DNA damage tolerance was also shown to be independent of Pol ζ in avian cells, where its absence exacerbated the phenotypes of Pol η/Pol ζ knockout cells (138). Notably, complementing PrimPol^−/−^ cells with a primase defective, but polymerase/TLS active, PrimPol did not rescue their damage sensitivity, further supporting the notion that PrimPol's primary role *in vivo* is to reprime DNA synthesis (130,138). These studies demonstrate how the existence of complementary damage tolerance pathways can mask the effects of losing one mechanism alone.

Another proposed DDT mechanism involves fork reversal following a major replisome stalling event. The breast cancer-associated (BRCA) proteins (BRCA-1 and BRCA-2) have been suggested to protect reversed forks from nucleolytic degradation and therefore promote bypass of DNA lesions by homologous recombination (148,149). Mutations in these genes are the leading cause of familial breast and ovarian cancers (150). As BRCA proteins protect replication forks undergoing reversal from degradation, BRCA null cell lines may be susceptible to increased genomic instability brought on by extensive fork degradation (55). It was reported that the fork degradation phenotype typically displayed by BRCA1 deficient cells after a single dose of cisplatin is absent after treatment with multiple doses, with cells exhibiting increased replication fork speeds (151). Overexpressing WT PrimPol, but not catalytically- or primase-inactive mutants, protected against the degradation phenotype observed in BRCA deficient cells. These results suggest that cells may upregulate their PrimPol-dependent repriming pathway in order to compensate for the loss of fork reversal as an alternative mechanism of cisplatin tolerance. Supporting this, following multiple cisplatin doses, PrimPol mRNA levels were significantly elevated and chromatin-bound PrimPol increased in BRCA-1 deficient cells, but not in cells complemented with BRCA-1. The increase in mRNA was found to be regulated by ATR. Another recent study reported that the USP36 protease also possibly plays a role in regulating PrimPol protein levels (152), and the ATPase WRNIP1 has been suggested to target PrimPol protein for degradation (153). This suggests that multiple mechanisms exist that regulate PrimPol deployment in human cells. This is probably not surprising as a failure to suppress ssDNA gaps has been suggested to be a major hallmark of BRCA-deficient cancers and a cause of their sensitivity to chemotherapeutic agents (154). Uncontrolled repriming may lead to a similar increase in ssDNA gaps, decreasing cell fitness unless cells can compensate, e.g. by increasing TLS activity as observed in some cancer cells (155).

Another study by Bai *et al.* (156) investigating cells deficient in the fork remodeller HLTF reached similar conclusions to those of Quinet *et al.* (151). HLTF is an SWI/SNF family chromatin remodelling enzyme that promotes fork reversal and, in its absence, PrimPol is required to maintain efficient fork progression, leading to the accumulation of ssDNA gaps (157,158). PrimPol's action at the fork appears to confer replication stress resistance and allow S phase to continue without slowing of DNA synthesis. However, if HLTF is present and allowed to bind to the replication fork but contains an inactive HIRAN domain (the domain that binds the 3′-hydroxyl group of nascent DNA), PrimPol is outcompeted at the fork and does not act, and the role of S phase progression is undertaken by Rev1. Similar work has shown that in the absence of CARM1, a protein implicated in the stabilisation of reversed forks, PrimPol and TLS are both employed in restarting replication forks (159). The balance between these complementary DTT pathways therefore has the potential to mask the key roles that repriming undertakes *in vivo*.

### Repriming is not the end of the story...

In eukaryotes, PrimPol's repriming activities allow replication to continue past lesions, structures and other impediments. However, once synthesised, the primer is likely to be some distance away from the CMG complex due to helicase uncoupling that accompanies leading-stand fork stalling (160). In order to restore efficient canonical replication, synthesis must be recoupled to the CMG. In the case of TLS, Pol δ conducts leading-strand synthesis following lesion bypass, which fits well with the previous reports of Pol δ replicating both strands after replication restart (9,10). PrimPol interacts with, and is stimulated by, Polymerase δ-interacting protein 2 (PolDIP2), which may facilitate a handoff from PrimPol to Pol δ, once primer synthesis is complete (136,161,162). Pol δ could then synthesise until replication can be recoupled to CMG-Pol ϵ, as is the case following TLS (9,163).

One of the major distinctions between the two human primases is that Pri1 produces an RNA primer, while PrimPol synthesises a predominantly DNA polymer. This preference to reprime using dNTPs may facilitate a more efficient restarting of DNA synthesis as high fidelity replicases preferentially copy B-form DNA templates, whereas priming with an RNA polymer produces an RNA–DNA hybrid that is a much poorer A-form substrate. In addition, it also eliminates the requirement for Pol α-dependent synthesis prior to primer handover to Pol δ/Pol ϵ. DNA primers may also be preferred to avoid introducing breaks on the leading strand. Following replication, RNA primers are subsequently excised (e.g. Fen1/Pol I) and then replaced with DNA to maintain genome integrity. However, during PrimPol-dependent repriming, it is likely that dNTPs are preferentially incorporated to prevent the processing and removal of the newly synthesised primers as this could result in undesirable strand breaks that are particularly dangerous on the leading strand. Notably, PrimPol incorporates a single initiating 5′ ribonucleotide during primer synthesis but this is likely removed by the RNase HII pathway in a post-replicative fashion (127,164).

Reinitiating DNA synthesis from a downstream primer generates a single-stranded gap in the nascent chain opposite the unresolved stalling lesion or structure. To complete replication, gaps must be filled in a manner that is independent of the global genome replication process. Interestingly, this event was not found to occur in any known DNA repair centres; instead, the majority of ssDNA accumulates in post-replicative repair territories (PORTs) (165). Here, TLS or template-switching pathways facilitate the removal of the ssDNA gaps. TLS does not solely occur directly at an active fork - following the monoubiquitination of PCNA, TLS enzymes can synthesise over bypassed UV lesions in a post-replicative manner to fill gaps that result from repriming (166). A recent study has also implicated homologous recombination in the gap-filling process at PrimPol-mediated ssDNA gaps in human cells (167). As part of this process, MRE11 and EXO1 facilitate 3′ to 5′ resection of DNA gaps to expose sufficient ssDNA for the loading of the HR protein, Rad51. Subsequent template switching allows the replication of the gapped region by using the unimpaired sister chromatid as a template. Presumably, persistent fork-stalling DNA structures must be resolved using the canonical mechanisms before opposing gaps can be filled.

### Coping without a bespoke repriming pathway

Since repriming represents a major canonical DDT pathway in most cells, how do organisms without specialised repriming mechanisms (e.g., budding/fission yeast, drosophila, *C. elegans*) deal with leading strand stalling events? As discussed, in prokaryotes this appears to be resolved by simply repurposing the replicative primases to also reinitiate replication. However, evidence to suggest that a similar process occurs in eukaryotic organisms is lacking. It is very likely that cells without a bespoke repriming pathway may simply rely on alternative DDT pathways and mechanisms to ultimately maintain genome stability without the requirement to conduct leading strand repriming. One such viable alternative pathway involves using TLS and, in fact, the budding yeast replisome has been shown to efficiently utilise Pol η to bypass leading strand lesions ‘on the fly’ (9). Additionally, recent findings indicate that the yeast replisome is itself inherently tolerant of oxidative damage (168). Upon encountering a leading strand thymine glycol or 8-oxo-G, Pol ϵ is switched for Pol δ, which conducts rapid, error-free synthesis over the lesion. There is also some evidence to potentially support a similar role for Pol δ in higher eukaryotes, although this mechanism remains to be established (169–171).

In addition, other functionally overlapping DDT pathways could also offer sufficient protection against the deleterious effects of leading strand lesions. For example, yeast cells display an abundance of recombination intermediates associated with fork reversal and temple-switching, in fitting with the preferential usage of recombination pathways by these organisms (reviewed in (172)). It is therefore likely that HR and other DDT pathways (e.g TLS, dormant origin firing) readily compensate for the lack of repriming mechanisms and these alternative mechanisms may even provide more efficient replication restart solutions for some organisms.

## CONCLUDING REMARKS

Since the seminal studies of Rupp and Howard-Flanders, the precise nature of leading strand synthesis has been debated (82). The initial evidence pointed to a mechanism whereby all DNA was synthesised in small pieces, regardless of which strand, leading or lagging, the nascent chain originated. A plethora of conflicting publications made it difficult to draw concrete conclusions and it seems the field gravitated towards the mechanistically simpler model of continuous leading strand synthesis from origin to termination. The discovery of TLS enzymes made the continuous argument even more appealing, as these enzymes offered an explanation as to how lesions could be bypassed without breaking the continuous nascent chain (89–91). However, the idea of repriming was not forgotten and later studies provided some compelling evidence of leading strand repriming occurring in bacteria and yeast (88,93,94). The discovery and subsequent characterisation of a second primase enzyme in vertebrate cells (PrimPol) has now established that a similar process also occurs in most eukaryotic cells and represents a key additional DDT pathway for maintaining efficient fork progression (161). With more research being conducted into leading strand repriming, it is becoming apparent that it offers a flexible replication restart pathway that is an ideal solution for bypassing a wide variety of fork stalling impediments, that can subsequently be resolved in a post-replicative manner. This is most apparent in studies that demonstrate functional redundancies between repriming and a variety of specific pathways for tolerating damage (126,139). Thus, after examining all of the available evidence, it is clear that repriming on the leading strand should now be considered a canonical DDT pathway in a wide range of organisms, from bacteria to human cells. In fact, repriming may even represent the original DDT pathway as it is also required to maintain efficient DNA duplication during unperturbed replication.

Although the significance of repriming mechanisms in vertebrate cells is becoming more evident, much remains to be discovered about this process. Since aberrant priming on ssDNA is clearly undesirable, there are likely many undiscovered regulatory mechanisms to ensure that the usage of repriming pathways is strictly restricted to when and where they are required. Additionally, taking into account the diverse range of functions displayed by other Prim-Pol superfamily members (103), it seems plausible that PrimPol may undertake additional roles in DNA replication and repair, e.g. TLS or gap repair synthesis. Since PrimPol is involved in an important mechanism that maintains replication restart in human cells, defects in this pathway are likely to have a role in genetic diseases. A specific PrimPol mutation has already been identified as a susceptibility gene for high myopia (173), although its role in the development of this condition has not been established. Additionally, PrimPol alterations have been observed in cancers, with overexpression of PrimPol reported in glioblastoma and a point mutation identified in lung cancer (174,175). Interestingly, DNA primase (PRIM1) mutations have recently been linked to the development of Microcephalic Primordial Dwarfism (MPD) (176), further demonstrating the association of primase mutations with disease states and highlighting the need for further research into the exact mechanisms that underpin this canonical DNA replication-associated restart pathway.

## DATA AVAILABILITY

No primary data is associated with this article.
